# Suicidal mothers

**DOI:** 10.5249/jivr.v3i2.98

**Published:** 2011-07

**Authors:** Salvatore Gentile

**Affiliations:** ^a^Department of Mental Health, Piazza Galdi, Italy.

## Abstract

**Background:**

Epidemiological research has demonstrated that suicidal ideation is a relatively frequent complication of pregnancy in both developed and developing countries. Hence, the aims of this study are: to assess whether or not pregnancy may be considered a period highly susceptible to suicidal acts; to recognize potential contributing factors to suicidal behaviors; to describe the repercussions of suicide attempts on maternal, fetal, and neonatal outcome; to identify a typical profile of women at high risk of suicide during pregnancy.

**Methods:**

Medical literature information published in any language since 1950 was identified using MEDLINE/PubMed, Scopus, and Google Scholar databases. Search terms were: "pregnancy", (antenatal) "depression", "suicide". Searches were last updated on 28 September 2010. Forty-six articles assessing the suicidal risk during pregnancy and obstetrical outcome of pregnancies complicated by suicide attempts were analyzed, without methodological limitations.

**Results:**

Worldwide, frequency of suicidal attempts and the rate of death by suicidal acts are low. Although this clinical event is rare, the consequences of a suicidal attempt are medically and psychologically devastating for the mother-infant pair. We also found that common behaviors exist in women at high risk for suicide during pregnancy. Review data indeed suggest that a characteristic profile can prenatally identify those at highest risk for gestational suicide attempts.

**Conclusions:**

Social and health organizations should make all possible efforts to identify women at high suicidal risk, in order to establish specific programs to prevent this tragic event. The available data informs health policy makers with a typical profile to screen women at high risk of suicide during pregnancy. Those women who have a current or past history of psychiatric disorders, are young, unmarried, unemployed, have incurred an unplanned pregnancy (eventually terminated with an induced abortion), are addicted to illicit drugs and/or alcohol, lack effective psychosocial support, have suffered from episodes of sexual or physical violence are particularly vulnerable.

## Introduction

A World Health Organization report has named depression as the greatest disease burden for women worldwide.^1^ In particular, childbearing years are a time of increased vulnerability to the onset or recurrence of major depressive disorder, thus placing young women at risk of suffering from severe affective impairment during pregnancy.^2,3,4^Indeed, antenatal depression is a significant public health problem. Information drawn from scientific literature underlines that, in developed countries, between 3% and 16% of pregnant women fulfill the diagnostic criteria for unipolar major depression.^5,6^In specific populations, such as marginalized minority groups and unmarried teenagers, the rate of clinically relevant mood symptoms in pregnancy may be as high as 51%.^[Bibr B7]7^In developing countries, the prevalence of antenatal depression is estimated at 33%.^8^

It is well-known that antenatal depression adversely impacts on several obstetrical, fetal, and infantile outcomes. In fact, untreated maternal mood symptoms have been associated with an increase in the rate of pregnancy complications (such as pre-eclampsia, premature delivery, impaired fetoplacental function, and low fetal growth) and perinatal problems.^9,10,11,12,13,14^

Maternal depression also induces significant effects on neonatal physiology. Elevated cortisol and norepinephrine levels, lower dopamine levels, and greater relative right frontal EEG asymmetry have all been described in newborns of mothers suffering from depression during pregnancy.^15,16^Findings from a preliminary, recent study^17^have also suggested that there is an elevated risk of lower birth weight among infants born to adolescent mothers who report depressive symptoms complicated by selfharm ideation and behavior compared to those born to adolescents who report depressive features without suicidal symptoms.

The detrimental impact of untreated maternal depression on the infant neurocognitive and psychological development is also well-known. Many untreated women continue to be depressed in the postnatal period and are at risk of impaired interaction with their infant at a time when the child is in a particularly sensitive developmental period,^18,19,20,21,22^and this may lead to poorer cognitive functioning and compromised social adaptation during childhood, adolescence, and young adulthood.^23,24,25^

Prevalently, however, depression is characterized by a dramatic increase in the risk of suicide or serious suicide attempts, since more than 90% of suicide victims have a diagnosable mental illness and approximately 60% of all suicides occur in persons with mood disorders.^26^

Suicide during pregnancy is included among the indirect causes of maternal death. The World Health Organization defines maternal deaths as all deaths occurring any time during pregnancy and up to 42 days after parturition.^27^Indirect causes of maternal deaths include all those conditions not directly related to obstetric cause but worsened by the pregnant status, such as complications due to cardiac disease and mental disorders. When all maternal deaths within one year after delivery are considered, suicide is one of the four leading causes of maternal deaths overall (together with thromboembolisms, obesity, and cardiac events).^28,29^ For this reason, maternal mortality associated with psychiatric illness should remain a focus of high clinical interest in the developed countries. In these countries, in fact, the rate of maternal deaths due to other conditions, such as poor antenatal care and malnutrition, have progressively decreased during the last century.^30^Moreover, the epidemiological studies have been mainly focused on the increased risk of suicidal attempts during the puerperal period, since suicides account for up to 20% of postpartum deaths.^31^

Hence, the primary aim of this study is to assess whether or not pregnancy may be considered a period highly susceptible to suicidal events. Other scopes of this review are: to recognize potential contributing factors to suicidal behaviors; to describe the repercussions of suicide attempts on maternal, fetal, and neonatal outcomes; to identify a typical profile of women at high risk of suicide during pregnancy, in order to establish effective strategies of prevention. 

## Methods

Medical literature information published in any language since 1950 was identified using MEDLINE/PubMed, Scopus, and Google Scholar databases. Additional references were identified from the reference lists of published articles. Search terms were: “pregnancy”, (antenatal) “depression”, “suicide”. Searches were last updated on 28 September 2010. Selected on the basis of their abstract or the full-text article when the abstract was unavailable, all articles assessing the suicidal risk during pregnancy and obstetrical outcome of pregnancies complicated by suicide attempts were acquired and analyzed, without methodological limitations. Forty-six articles met the wide inclusion criteria. The Author was the only reviewer who performed both data selection and extraction. 

## Results

**Prevalence of suicidal ideation in pregnancy**

In the Western world, recent epidemiological researches have demonstrated that suicidal ideation may be detectable in a percentage ranging from 13.1% to 33.0% of pregnant women.^32,33^Symptoms frequently associated with suicidal ideation include depressed mood, lack of concentration, anxiety, preoccupations, obsessive compulsive symptoms, tiredness, worry concerning bodily functions, and compulsion.^34^

In developing countries, too, nearly 14% of pregnant women admit to thoughts of self-harm.^8^In these regions of the world, the occurrence of suicidal ideation during the gestational period is a relevant concern especially among adolescents, since it has been reported in up to 17% of young mothers.^35^

**A. How many mothers die by suicide?**

**Europe**

A Danish register/case audit study based on data from the registers of the Danish Medical Health Board, death certificates, and hospital records covering the period 2002-2006 identified 26 cases of maternal death, leading to a maternal mortality ratio of 8.0/100,000 live births.^36^No cases of death due to suicide were identified. Similar results derived from data extrapolated from three national Finnish health registers^37^and two Swedish national registers.^30^

Only five cases of suicidal deaths during pregnancy have been described in the United Kingdom report Why mothers die 2000-2002.^38^In three of these cases, the women had been diagnosed with severe form of psychiatric disorders, such as major depression (two cases) or schizophrenia (one case).

**North America**

An epidemiological U.S. study performed on a large State of California database showed that the cumulative incidence of suicide attempts during pregnancy was four per 10,000 pregnancies.^39^The contribution of several injuries to maternal mortality was assessed through a review of New York City medical examiner records of 2331 women 15-44 years of age who died of injuries during 1987-91.^40^The largest proportion of injury-related deaths were homicides (63%), whereas suicide accounted for only 11 cases. To further determine the prevalence of suicide during pregnancy, the autopsy reports of all female residents of the same city, 10-44 years old, who committed suicide from 1990 to 1993 were assessed for pregnancy.^41^The standardized mortality ratio for suicide during pregnancy was 0.33; that is, the number of suicides of pregnant women was only one-third of that expected.

A retrospective study of abortion and maternal deaths in Minnesota during the period 1950-1965 including data on more than 1,400,000 women reported only four cases of suicides that occurred during the gestational period,^42^whereas the Maternity Mortality Collaborative (a U.S. program focused to investigate incidence, risk factors, and causes of maternal deaths in 19 regional areas) identified 13 cases of suicide among 712 deaths of women during pregnancy that occurred between 1980 and 1985.^43^

In the year 2000, a collaborative effort involving World Health Organization (WHO), UNICEF, and UNFPA estimated 660 maternal deaths in the United States. This averages out at 11 maternal deaths per 100,000 live births reported.^44^The most common causes of maternal death varied somewhat from region to region. Several medical and obstetric complications, but not suicidal attempts, were identified as significant contributors to maternal mortality in these populations. Moreover, only five cases of maternal suicidal deaths were identified through a follow-up investigation of 41 injury-related maternal deaths identified from 1992 to 1994 in North Carolina.^45^Canadian data also excluded a significant risk of suicidal death during pregnancy.^46^

**Asia**

An epidemiological study^47^looked at maternal deaths in Singapore from 2000 to 2004, by linking coronial cases of female suicide in the reproductive age group 15-45 years, with the birth registration database, to identify both early and late maternal deaths. Among the 589 cases of identified maternal death due to suicide, none of these occurred during pregnancy.

**Africa**

Similar results emerged after analyzing data from African countries. An estimation of maternal mortality rates for 1982-83 in Zambia led to identify just one case of suicide (induced by an illegitimate pregnancy).^48^Of 175 maternal deaths reported in two district of Zimbabwe over two years (1989-1990), four were listed as suicide owing to unwanted pregnancy.^49^

**B.Factors contributing to increase the suicidal risk in pregnancy are summarized in Table 1.**

**Developing countries**

Suicide can be precipitated by an illegitimate pregnancy, especially in those societies where social sanctions and religious condemnation are particularly harsh.^50^Moreover, in specific religious and cultural contexts, conceiving a female fetus, history of being beaten by the husband either during or before the current pregnancy, and an unhelpful or unsupportive mother-in-law also show a strict connection with antenatal depression and suicidal behavior.^8^Explanations for these associations appear to be rooted in the culture of many developing countries. In traditional societies, where the wife’s mother-in-law is the matriarch who holds effective power and control and the daughter-in-law is under her strict guidance and supervision, a strong familial preference exists towards the birth of male infants.^51^(Table 1).

**Table:** Table 1: Contributing factors to suicidal behaviors in pregnancy*

Developing countries	Developed countries
Intimate partner violence, both physical and sexual	Intimate partner violence, both physical and sexual
Unmarried status	Unmarried status
Teen age #	Teen age #
Pregnancy outside marriage €	Unplanned pregnancy
Lack of effective familial and/or social government institution supports	Lack of effective familial and/or social government institution supports
Conception of a female fetus, especially in those traditional societies where a strong familial preference exists towards the birth of male infants	Previous or present history of any psychiatric disorder
Poor mother-in-low support	Street drug/alcohol abuse
	Unemployment
	Induced abortion

* These findings are not in a ranking numeric order # Age ranges are not available € Pregnancy in unmarried women

**Developed countries**

The rate of suicidal ideation in pregnancy is significantly associated with psychiatric disorder and, especially, with current major depressive episode and comorbid anxiety disorder and/or substance abuse.^8,32^Psychosocial factors which may also contribute to increase the rate of maternal suicide attempts during pregnancy are teen age, unplanned pregnancy, unmarried status or recent divorce, unemployment, and difficult access to safe abortion service.^51,52^Moreover, hospital-based, cohort studies, and literature reviews have suggested that intimate partner violence, previous experience of sexual assaults, interpersonal conflicts also represent specific factors associated with an increased suicidal risk.^53,54,55^Experiencing intimate partner violence may increase the risk of suicidal behaviors by enhancing the risk of impulsive responses and facilitating the onset of posttraumatic stress disorder, since both situations are intrinsically associated with an increased suicidal risk.^56,57^In addition, both severe emotional distress^58^and overall suicidal risk is increased after voluntary pregnancy termination. Indeed, the risk of suicidal ideation and attempt is at least doubled in women who experience abortion;^59,60^though other studies^61^report that the suicide rate among women who have abortions may be as much as six times higher than that of women who have given birth in the prior year, and double that of women who experience spontaneous abortion. Of note, however, the strongest evidence-based confirmation regarding the relationship between voluntary pregnancy termination and an increase in the suicidal risk derives from a review available from a journal issued by a catholic association.^62^A possible explanation of the increased risk of suicidality after induced abortion is that this decision, for many women, rather than being a relief and/or an answer to their problems, may be additional proof of their worthlessness and may contribute to suicidality;^63^indeed, it has been reported that a post-abortion suicide attempt may be an impulsive act of despair.^64^

**C. Most frequently used methods for attempting suicide in pregnancy.**

Almost invariably,  pregnant women  choose poison  ingestion or drug overdose for their suicide attempts.^65^Indeed, a Thailand study demonstrated that poisoning is the commonest method of suicide, organophosphate pesticides such as parathion being the poison of choice.^66,67^

In contrast, in Western countries several licit drugs, such as benzodiazepines, are the most frequently used drugs for attempting suicide.^68^However, different medications, including analgesics (most notably acetaminophen), vitamins or iron, antibiotics, antihistamines or decongestants may be used in order to attempt suicide.^69^Moreover, despite the use of barbiturates being  progressively decreased in both neurological and psychiatric practice, such medications are still used by suicidal pregnant women.^70^The period of pregnancy at highest risk of suicide attempts seems to be the second trimester.^71^

**D. Maternal and neonatal outcome after attempted suicide.**

Interesting information comes from studies focused on investigating whether or not suicide attempts by drug overdose may impair obstetric, fetal, and neonatal outcomes. In answer to this question, women diagnosed with drug intoxication during pregnancy were identified in the Regional Hospital Discharge Registry of North Jutland from 1977 to 1999 by linkage of diagnoses for abortion and delivery with diagnoses for intoxication. Hospital medical records were reviewed to obtain data on drug use, dosage, and pregnancy outcome:^72^of 122 women studied, 17 experienced spontaneous abortion. Hence, in general, a drug overdose shortly before or during pregnancy might have been associated with a doubled risk of spontaneous abortion whereas, compared with the background population, there was no increased risk of congenital abnormalities or prematurity. No increase in the structural teratogenic risk was demonstrated in pregnant women who attempted suicide by specifically ingesting very large doses of different barbiturates.^71^Cognitive status and performances at behavioral scales of children exposed to barbiturates throughout placenta also did not indicate any developmental impairment.^71^

Recently, however, very large dose of specific benzo-diazepines (such as nitrazepam) used for suicide attempts during pregnancy was associated with increased rates of congenital birth defects (which may be connected with the disruption of protein metabolism in fetal mesenchyma).^73^Moreover, a linked Vital Statistics-Patient Discharge State of California database confirmed that suicide attempts during pregnancy might be associated with significantly higher rates of maternal and perinatal morbidity, and in some cases, perinatal mortality.^30^In fact, such women showed an increase in the risk of premature labor, caesarean delivery, and need for blood transfusion. Analysis of neonatal outcomes also revealed increases in respiratory distress syndrome and low birth weight.^38^In addition, maternal death due to suicide may seriously compromise the future development of orphans. In fact, children who lose their own mother because of suicidal acts show a greater risk of hospitalization for all types of psychiatric disorders and suicide attempts than offspring of parents who have died of other causes.^74^

## Discussion

Review data seem to suggest that prevalence of unipolar depression and suicidal ideation in pregnancy is relatively high,^5-8,32,33,35^However, frequency of suicidal attempts and, especially, rate of death by suicidal acts are low in both developed and developing countries.^36-49^Although rare, consequences of a suicidal attempt are likely to be devastating for the mother-infant pair.^36,72-74^

Moreover, local social and health organizations may consider such women unable to carry out sound maternal functions;^75^hence, infants who survive maternal suicide attempts during pregnancy could be at increased risk of institutionalization. For these reasons, social and health organizations should make all possible efforts to identify women at high suicidal risk, in order to actuate specific programs focused on preventing tragic outcomes. Suicidal literature allows to identify the typical profile of women at high risk of suicide during pregnancy. Hence, the first step towards preventing suicidal events in pregnancy is to identify pregnant women who are at elevated risk factors.^8^In developed counties, those women who have a current or past history of psychiatric disorder,^50^are young, unmarried, have incurred an unplanned pregnancy which has terminated in abortion,^51,52^are addicted to illicit drugs and/or alcohol, lack effective psychosocial support, have suffered from episodes of sexual or physical violence are particularly vulnerable.^8,53-55^

The second step of an effective prevention-plan - since between 4% and 8% of all women experience intimate partner violence during pregnancy^76^should consist of organizing specific social task-forces to implement and publicize (in both developing and developed countries and either in disadvantaged or privileged social context) specific programs which may facilitate women experiencing abusive relationships to withdraw from such perverse attachments.^8^Such programs should also involve those women who have temporarily chosen to continue the affective bonding of a desire to ensure a “normal” family to their children. Obviously, these programs must include both practical support (e.g., safe accommodation, decent work for unemployed women, and facilitated access to gynecologic care and legal assistance) as well as psychological support, finalized to facilitate the choice of continuing gestation.^77^Alternatively, in the case of an unwanted conception, easy access to public abortion services should be available to those women who choose to interrupt pregnancy.^8,52^

The third step is to promote close cooperation between community psychiatric services, family physicians, hospital gynecological units, and local social services in order to provide the best available care for all women diagnosed with psychiatric disorders who have become mothers. Regrettably, however, up to 70% of patients with mental problems withdraw psychotropic medication during pregnancy and refuse psychiatric counseling;^78^of note, care does not resume during the postpartum period.^79^Thus, such women should be informed, at least during early pregnancy, that the risk of untreated mental disorder outweighs the risk for the fetus of exposure to most psychotropic medications.^80^In fact, several classes of psychotropic seem devoid of significant structural teratogenic risks. Among antipsychotics, chlorpromazine is the drug which shows the highest number of studies suggesting its reproductive safety	^81^whereas, among antidepressants, fluoxetine and sertraline should be preferred, since both (anecdotally) have been associated with slight increase in fetal malformations.^80,82^It is also possible to safely start or continue some mood stabilizing agents, since the slight increase in the risk of orofacial cleft suggested for lamotrigine^2^has not been confirmed in recent studies.^83^However, nearly all these medications (with the exception of lamotrigine) have been associated with an increased risk of perinatal complications if used in late pregnancy.^84^Therefore, if clinicians deem indispensable the continuance of psychotropic medications during the last stage of the gestational period, they must firmly suggest that delivery happens in hospitals equipped with specialized neonatal intensive nursery units.^85^

However, the vast majority of maternal deaths due to suicide occur after parturition.^31^Thus, social, psychological, and clinical support provided to such mothers should be continued for at least the first six months of puerperium. The prolonged support may reduce the risk of other tragic events, such as neonaticide (child homicide on the first 24 hours of life) and infanticide (child homicide within the first year of life), which could be brought by mothers who develop antenatal depression and continue to be depressed after parturition.^86^The support resources should gradually be discontinued only when the personal and clinical situation of the mothers appears to be satisfactorily and definitively stabilized.

## Conclusions

Although reviewed studies do not allow us to differentiate between factors associated with suicidal ideation and factors which may contribute to the occurrence of violent and definitive suicidal acts, available data clearly indicate a noteworthy discrepancy between the prevalence of suicidal ideation during pregnancy (relatively high) and the rate of death due to intentional self-harm (extremely low). This discrepancy seems to suggest that maternal death due to suicide may occur merely in the presence of several, concomitant triggers which involve different areas of the complex maternal universe such as compromised mental stability, weakness of the desire to experience maternity, unhealthy life-style, poor affective liaisons and family support, disadvantaged economic conditions, difficulty in adherence to rigid cultural and religious rules (especially in contexts which stigmatize any female non-orthodox behaviors), and a previous history of personal traumatic experiences (including intimate partner violence and induced abortion).
